# Foreign Correspondence

**Published:** 1863-10

**Authors:** 


CHICAGO
MEDICAL JOURNAL.

VOL. VI.] OCTOBER, 1863. [No. 10.

### ORIGINAL COMMUNICATIONS.

### FOREIGN CORRESPONDENCE.

Bagneres de Luchon, August 24th, 1863.

*My Dear Doctor:*—This place is noted for its antiquity, it having been inhabited by the Romans; for the picturesque beauty of its location; situated in a circumscribed valley, surrounded by lofty mountains; so near the source of the Garonne as to be called with much propriety "the Heart of the Pyrenees." It is now one of the fashionable resorts of the French, especially, owing to the repute in which its waters are held for their therapeutic virtues.

France is probably more liberally supplied with mineral waters than any other portion of the continent; the springs being reckoned at many hundreds, issuing from the earth in various localities, the larger portion being met with in the Pyrenees.

The mineral elements vary greatly, not only in the waters of different localities, but also in the different springs of the same station, and the proportion of these elements differ likewise in the waters of each spring from those of all the others, no two being exactly alike in composition. The temperature varies from very cold to very hot, ranging so high as 150° or 170° Far.

The waters of all these springs have been classified as follows: 1st. Sulphurous; 2nd. Ferruginous; 3d. Alkaline; 4th. Gaseous; 5th. Bromo-iodurets; 6th. Saline. Most of these waters have been carefully, and doubtless *accurately*, analyzed, but this cannot with confidence be affirmed of all, as the following piquant and instructive incident will illustrate:

The waters of Neyrac were reported by M. Mazade to be of great therapeutic value, as he asserted he had discovered that they were rich in valuable minerals, containing among others the following: Molybdenum, tungstein, cerium, cobalt, titinium, &c., &c., sufficient to render them of greater value than any other known mineral waters. The Academy of Medicine appointed a commission to verify the assertion; the committee reported favorably, and the Academy thereupon awarded a prize medal. Some time subsequently M. LeFort, a pharmaceutist of Paris, reported to the Academy that he had proved, by a rigid analysis, that these springs of Neyrac did not contain a single element claimed for them.

The maladies for which the waters of the Pyrenees are recommended include almost the entire list of diseases known to modern practice; as paralysis, neuralgia, amaurosis, phthisis, bronchitis, asthma, cardiac disease, the innumerable diseases of the digestive organs, the various diseases of the genito-urinary system. They are prescribed with great confidence in many uterine afflictions; the physicians at the various stations assert as proof of the value of the springs in this class of cases that engorgement of the neck of the uterus, ulceration, &c., are speedily cured; and their efficacy in prolapsus uteri is incontestible, as the patients very soon lay aside their pessaries after using the waters! all of which is (equivocally) confirmed by the patients themselves, for they frequently find the treatment so beneficial, that they return some six, eight or ten years in succession!! Can more proof be needed?

Educated physicians reside at all the stations; some one or more of whom are appointed by the Government as Inspectors, who direct what bath the patient shall take, the length of time he shall remain in, and all the minor particulars, the quantity of water he shall drink each day, &c. *Theoretically* it is necessary to have the prescription to enable the stranger to obtain a bath, but *practically*, not, for I find no difficulty at any time.

The bath is taken by invalids once a day,either of Sulphurous water, Ferruginous, or the two mixed in variable proportions to suit the individual case, according to the notion of the Inspector. The hot douche is considerably used, in local diseases; and a large quantity of the water is taken internally by each patient daily. The *douche* is applied to all cases of paraphlegia, and an intelligent gentleman from New York, with incipient amaurosis, is having the hot water applied in this manner to his eyes and temples. In response to an enquiry, he expressed some hope of receiving benefit from the treatment, as he already felt a little worse! It is given out by the physicians at the different stations, and believed by the patients that the symptoms of their particular case must be aggravated for a certain time; and then they may expect a sudden change for the better, which is to continue till they are restored! thus I suppose they remove disease by exciting an artificial crisis.

There can be no doubt, I think, that some diseases are alleviated and others cured by the means thus resorted to, as certain cutaneous diseases, chronic rheumatic affections, &c., when the waters are well chosen and judiciously applied. But that all the efficacy that is attributed to these springs, is to be generally experienced, is more than we have reason to expect.

It seems to be a general fashion for the physicians of Paris and other large towns in France, to send their chronic cases to some one or other of the numerous watering places during the "season," which extends from the 1st of July to September. At least one thing is gained by this practice, the attending physician is relieved from the annoyance of a troublesome case, for a time.

Some idea of the extent to which the watering places of France are patronized may be obtained by taking Luchon as an example. The bathing establishment is a building 300 feet long, and about 100 feet wide, affording means for a large number of baths at the same time. The baths are prepared as early as 4 o'clock, A. M., and continue in requisition till late in the evening. The number of visitors here during the season will not vary far from six thousand.

An early walk from the Hotel to the bathing establishment is not without interest, from the contrasts that are presented, for we meet crowds who are wending their way to the "bath" or returning to their lodgings, and may see the thin-faced, sallow, sour-looking, confirmed valetudinarian, the wide-brimmed priest, the Parisian exquisite, stooping age, and beauties of the gentler sex, with a slight sprinkling of the English and American element, all pilgrims to the temple of Hygeia.

The first snow of the season fell on the night of the 20th inst., covering the tops of the nearest mountains to a considerable depth. M.

